# Pharmacological Potential of the Endogenous Dipeptide Kyotorphin and Selected Derivatives

**DOI:** 10.3389/fphar.2016.00530

**Published:** 2017-01-12

**Authors:** Juliana Perazzo, Miguel A. R. B. Castanho, Sónia Sá Santos

**Affiliations:** Instituto de Medicina Molecular, Faculdade de Medicina da Universidade de LisboaLisboa, Portugal

**Keywords:** kyotorphin, blood–brain barrier, kyotorphin-derived peptides, drug candidates, biological effects, clinical application

## Abstract

The endogenous peptide kyotorphin (KTP) has been extensively studied since it was discovered in 1979. The dipeptide is distributed unevenly over the brain but the majority is concentrated in the cerebral cortex. The putative KTP receptor has not been identified yet. As many other neuropeptides, KTP clearance is mediated by extracellular peptidases and peptide transporters. From the wide spectrum of biological activity of KTP, analgesia was by far the most studied. The mechanism of action is still unclear, but researchers agree that KTP induces Met-enkephalins release. More recently, KTP was proposed as biomarker of Alzheimer disease. Despite all that, KTP limited pharmacological value prompted researchers to develop derivatives more lipophilic and therefore more prone to cross the blood–brain barrier (BBB), and also more resistant to enzymatic degradation. Conjugation of KTP with functional molecules, such as ibuprofen, generated a new class of compounds with additional biological properties. Moreover, the safety profile of these derivatives compared to opioids and their efficacy as neuroprotective agents greatly increases their pharmacological value.

## Discovery, Distribution, and Receptors

The endogenous dipeptide L-tyrosine-L-arginine (YR) was first isolated from bovine brain in 1979 and found later on in other mammals’ brains and in human cerebrospinal fluid (CSF) (Takagi et al., 1979b; Ueda et al., 1980; Nishimura et al., 1991; Santos et al., 2013). The dipeptide with endorphin-like properties discovered in Kyoto was named kyotorphin (KTP) (Takagi et al., 1979a).

Kyotorphin can be formed in the brain by two pathways: (i) from precursor proteins degradation either by membrane-bound aminopeptidase or cytosolic Ca^2+^ activated protease (Ueda et al., 1985; Yoshihara et al., 1990; Akasaki et al., 1995); and/or (ii) from its precursor L-amino acids, tyrosine and arginine, in a reaction catalyzed by KTP synthetase dependent of ATP and Mg^2+^ (Ueda et al., 1987). This pathway produces three–fourfold more KTP than the one formed by precursor proteins degradation (Ueda et al., 1987).

KTP synthetase distribution correlates closely with KTP levels in the rat brain (Ueda et al., 1987). Its enzymatic activity was also detected in rat adrenal glands and spinal cord (Kawabata et al., 1996).

KTP is unevenly distributed over the brain. Lower brain stem regions, such as midbrain, pons, medulla oblongata and dorsal part of spinal cord are KTP-rich regions. These regions are sensitive to morphine and/or electrical stimulation-induced analgesia. However, 50% of total KTP brain’s content is concentrated in cerebral cortex, an area with low content of opioid receptors and enkephalinases (Ueda et al., 1980). Lower KTP contents can be found in striatum, hippocampus, hypothalamus, thalamus, and cerebellum (Ueda et al., 1980). The regional distribution of KTP supports the idea that KTP might have non-opioid actions.

Subcellular fractioning revealed KTP is enriched in synaptosomal fraction. Synaptosomes preloaded with KTP were able to release the dipeptide in a Ca^2+^ dependent manner upon depolarizing stimuli and it seems KTP can be recaptured again by synaptosomes in a Na^+^, temperature and energy-dependent manner. These data support the idea that KTP plays a role as neurotransmitter/neuromodulator (Ueda et al., 1986a,b).

Specific binding-assays using radioactive KTP suggest the presence of high affinity and low affinity receptors in the brain. The mechanism triggered by the binding of KTP to KTPr is mediated through protein *G*_i_ and phospholipase C (PLC) system that induces Ca^2+^ influx (Ueda et al., 1989). Thus, a nerve impulse is generated, ultimately leading to analgesia. The synthetic dipeptide L-leucine-L-arginine (LR) also binds KTPr with a great affinity but no effect, thereby constituting a potent antagonist (Ueda et al., 1989).

Interestingly, extremely low doses of KTP (femtomolar range) and more stable analogs (atomolar range) administered peripherally elicit nociceptive responses due to the release of substance P (SP) by nociceptor endings of primary afferent neurons. The authors speculate that the mechanism involved in such opposite responses (antinociceptive vs. nociceptive) might be mediated by different G proteins depending on KTP dosage, having a differential effect on PLC activation (Inoue et al., 1999; Ueda and Inoue, 2000).

The putative KTPr has not been identified yet. Despite many papers refer to “the KTPr” (Ueda et al., 1980, 1986b, 1989, 2000; Ueda and Inoue, 2000), it is not clear whether the receptor is specific or formed by μ- and δ-opioid receptors oligomerization (Machuqueiro and Baptista, 2007). From previous studies it is known that KTPr binding pocket must be different from opioid receptors (Takagi et al., 1979b; Rackham et al., 1982; Ueda and Inoue, 2000), but conformational studies on KTP suggested they should be structurally similar (Machuqueiro and Baptista, 2007).

## Metabolism and Clearance

### PEPT2 Transporter

Neuropeptides are released in the brain to exert their function and thereafter they are cleared either by extracellular peptidases and/or removed from extracellular fluid by specific transporters. Both processes have shown to be equally important in KTP clearance (Xiang et al., 2010).

Fujita et al. (1999) were the first to report an interaction between KTP and the high-affinity transporter PEPT2, albeit in an indirect way, evaluating competitive inhibition of glycylsarcosine (GlySar) in rat synaptosomes. Later, KTP uptake by PEPT2 was demonstrated in a more direct way, measuring the peptide-induced inward currents (Thakkar et al., 2008).

PEPT2 is high-affinity and low capacity transporter which rely on a pH gradient between extracellular and intracellular compartments to transport di- and tri-peptides. This transporter is expressed in kidney, retina and brain (Liu et al., 1995; Berger and Hediger, 1999). Hybridization studies showed PEPT2 is expressed in astrocytes and ependymal cells throughout the brain, and also in epithelial cells of choroid plexus (Berger and Hediger, 1999; Dieck et al., 1999). Astrocytes are essential in the control of neuronal activity and synaptic neurotransmission (Araque et al., 1999), and several peptidases are known to be expressed on their extracellular membrane (Berger and Hediger, 1999). Therefore, PEPT2 function in astrocytes might be linked to the removal of neuropeptide fragments and small biologically active peptides from extracellular fluid, such as KTP and carnosine (Berger and Hediger, 1999). In choroid plexus, the transporter is specifically located in the apical membrane, suggesting a role in the efflux of peptides from CSF (Shu et al., 2002). In addition, PEPT2 null mice showed enhanced antinociceptive response to intracerebroventricular (i.c.v.) administered KTP (Jiang et al., 2009). Transport of peptides from blood to CNS via PEPT2 is unlikely since this transporter is not present at blood–brain barrier (BBB) (Berger and Hediger, 1999).

### KTP-Degrading Aminopeptidases

Kyotorphin-degrading aminopeptidase activity was reported in brain homogenate (Ueda et al., 1985), lung and skin from rats (Orawski and Simmons, 1992). KTPase activity was inhibited by bestatin, but not puromycin, a potent inhibitor of soluble aminopeptidases (Ueda et al., 1985). Akasaki and Tsuji (1991) and Akasaki et al. (1991) identified two distinct KTPases in soluble fraction of rat brain. KTPase I is responsible for 95% of KTP-degrading activity, while KTPase II, which showed to be an enkephalin aminopeptidase, contributes only to 5% of KTP degradation.

Moreover, other authors found two dipeptide-cleaving enzymes associated to synaptic membranes (Orawski and Simmons, 1992). Both enzymes are inhibited by bestatin, but they can be distinguished based on their differential sensitivity to amastatin (Orawski and Simmons, 1992). Although KTPase I, described in Akasaki et al. (1991) presents similar characteristics to the KTP-degrading enzyme found by Orawski and Simmons (1992), it is not clear if they are identical (Akasaki et al., 1995).

## Mechanism of Action

The first experiments done by Takagi et al. (1979b) revealed an analgesic activity of KTP 4.2 fold more potent than met-enkephalins, when injected intracisternally; an effect that was reversed by naloxone. Although naloxone is an opioid antagonist, studies demonstrated KTP itself does not bind to opioid receptors, but has rather an indirect action mediated by met-enkephalin and β-endorphin, which activate δ- and/or μ-opioid receptors (Takagi et al., 1979b; Rackham et al., 1982; Ribeiro et al., 2011a). Other authors confirmed KTP-induced met-enkephalin release from guinea pig and rat brain slices (Shiomi et al., 1981; Takagi et al., 1982; Janicki and Lipkowski, 1983). In addition, there are evidences that KTP can inhibit some enkephalinases. This implies that met-enkephalins would be more protected from enzymatic degradation resulting in a relatively long-lasting analgesia (Takagi et al., 1979b; Hazato et al., 1986).

In patients with persistent pain, L-Arg intravenously administered induced analgesia and this action was antagonized by naloxone (Takagi, 1990; Takagi et al., 1990; Harima et al., 1991). It has been postulated that L-Arg can facilitate KTP synthesis in the brain, enhancing met-enkephalin release, which in turn activate δ- and/or μ-opioid receptors resulting in antinociception (Kawabata et al., 1993).

Alternatively, L-Arg is a well-known substrate for NOS. NOS and soluble guanylyl cyclase inhibitors administered i.c.v. caused antinociception. These findings led Kawabata et al. (1992, 1993) to conclude that L-Arg plays a dual role in nociceptive processing. In this case, NO-cyclic guanosine monophosphate (cGMP) pathway seems to be involved in nociceptive promotion in the CNS.

Kyotorphin-synthetase activity was detected outside the central nervous system (CNS), in adrenal gland, suggesting that KTP might have a role in peripheral system (Kawabata et al., 1996). In accordance, a study conducted in brown fat cell culture system showed KTP inhibited cell proliferation induced by noradrenaline. This indicates that these cells and probably other tissues in the periphery contain receptors for KTP (Bronnikov et al., 1997). In order to evaluate if KTP analgesic effect in the periphery was mediated by opioid receptors, a peripheral pain reflex test was conducted in mouse. Results showed the mechanism was mediated via KTPr but independent of opioid receptors, since naloxone was unable to prevent KTP analgesic effect (Inoue et al., 1997). The mechanism of action of KTP still not clear and some data are contradictory. However, there seems to exist two distinct pathways leading to analgesia, one mediated by opioids and other opioid-independent.

## KTPwas clustered in classes according to regular intervals of amplitude of 100 pg/mL Beyond Analgesia

### Physiological Effects

In addition to the extensively studied analgesic effect, KTP gathers a wide spectrum of biological activities (Dzambazova, 2010). Published papers have explored KTP role as antiepileptic (Godlevsky et al., 1995), thermoregulator (Sakurada et al., 1983), anti-hibernation regulator (Ignat’ev et al., 1998) and stress (Summy-Long et al., 1998) and behavior (Kolaeva et al., 2000) modulators.

Kyotorphin is present at moderate concentrations in the hypothalamus (Ueda et al., 1980), a structure with an important role in thermoregulation and stress. Naloxone-irreversible hypothermia was induced in mice, at room temperature, after i.c.v. administration of KTP and a more stable analog. However, thyrotropin (TRH) prevented this effect, suggesting the mechanism of KTP thermoregulation in the brain might involve the TRH neuronal system instead of opioid receptors (Sakurada et al., 1983). Regarding stress, high doses of KTP injected i.c.v. presumably activates the sympathetic nervous system, inducing a release of oxytocin (OT), a stress-hormone in rodents, concomitantly with elevating blood pressure and glucose plasma levels, but not vasopressin (Summy-Long et al., 1998).

Behavioral studies in rats and goldfish showed KTP reduced exploratory behavior mediated probably by the monoaminergic brain systems (Kolaeva et al., 2000).

### KTP as a Promising Biomarker in Alzheimer’s Disease

Recent estimates indicate 35.6 million people worldwide affected by dementia, a number that is expected to nearly double every 20 years (WHO, 2012). Alzheimer’s Disease (AD) is the most prevalent form of dementia in later life. It is clinically characterized by a progressive deterioration of memory, orientation, language, learning capacity, emotional stability, motor skills, and ultimately self-care, causing social and occupational disability. Unfortunately, no effective cure is available and current treatment strategies only provide symptomatic relief without halting nor reversing disease progression.

There is an increased need for AD biomarkers to improve early detection, accurate diagnosis, and accelerate drug development in this field (Flaten et al., 2006; Hampel et al., 2010). In medicine, a biomarker is generally defined as a molecule or any other tangible parameter that serves as an indicator of biological or pathogenic processes that can be used to evaluate disease risk or prognosis, and to monitor therapeutic interventions (Hampel et al., 2010). Decreased CSF levels of β-amyloid peptides (Aβ_40_, Aβ_42_) combined with increased levels of total tau and phosphorylated tau proteins, have diagnostic value in AD (Flaten et al., 2006; Hampel et al., 2010), for instance.

Although chronic pain is also highly prevalent in AD patients (Pieper et al., 2013), its proper evaluation and treatment is a clinical and ethical challenge. It may seem strange because AD patients consume fewer analgesics than other patient groups, making the false impression that they may feel less pain. However, processing and perception of pain are not diminished in AD (Cole et al., 2006). In those patients, pain underestimation relates with their limited capacity of verbally expressing their pain or discomfort, worsening as the dementia progresses (Pieper et al., 2013). Recent evidence suggest that chronic pain contributes to the course of neurodegenerative events as an additional injury to the nervous system (Borsook, 2012). Cognitive impairment and limited communication in AD patients leads to underreported pain, which in turn leads to undertreatment. By failing to receive adequate pain treatment, structural and irreversible changes may occur in the nervous system, aggravating AD pathophysiology which in turn contributes to chronic pain, maintaining a vicious cycle.

The benefits of finding a particular AD biomarker involved in the overlapping mechanisms of nociception and neurodegeneration that can be used in the clinics alongside with the existing ones (Flaten et al., 2006; Hampel et al., 2010) are obvious: (a) possibility to evaluate pain, regardless the level of cognitive impairment of the patient; (b) a biomarker that is itself a promising strategy for pharmaceutical development.

Our recent clinical studies showed a link between pain, AD and KTP in humans. In fact, we observe that pain was underestimated in AD patients (Santos and Castanho, 2013) and KTP has decreased levels in the CSF of AD patients with moderate cognitive impairment (Santos et al., 2013). Several other neuropeptides have been identified as diminished in AD (Raskind et al., 1986; Albericio et al., 1990). Lower levels of an analgesic molecule such as KTP may probably explain why AD patients are believed to have a higher incidence of hidden chronic pain; in agreement, Nishimura et al. (1991) have shown that CSF KTP levels decrease in chronic pain conditions.

**FIGURE 1:**
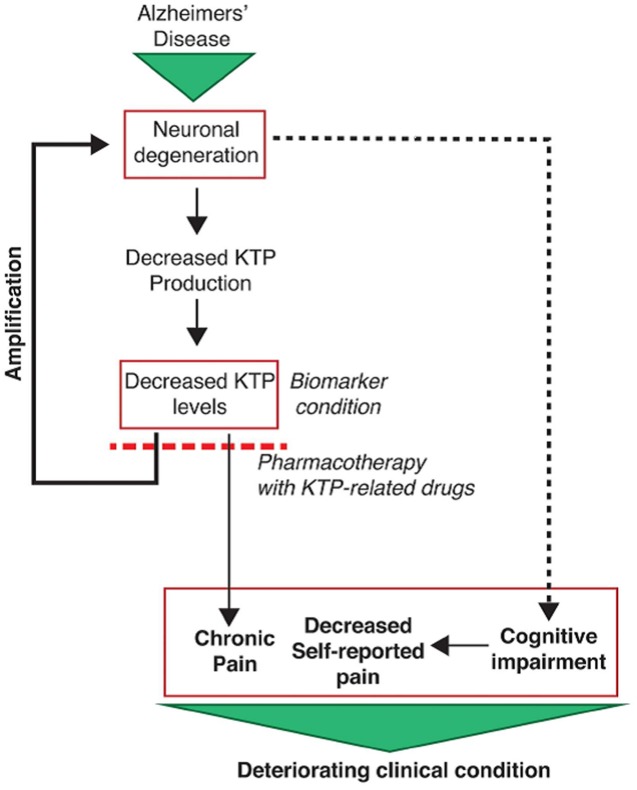
**Clinical implications of reduced KTP levels in cerebrospinal fluid (CSF) of Alzheimer disease (AD) patients (Santos et al., 2013)**.

**FIGURE 2:**
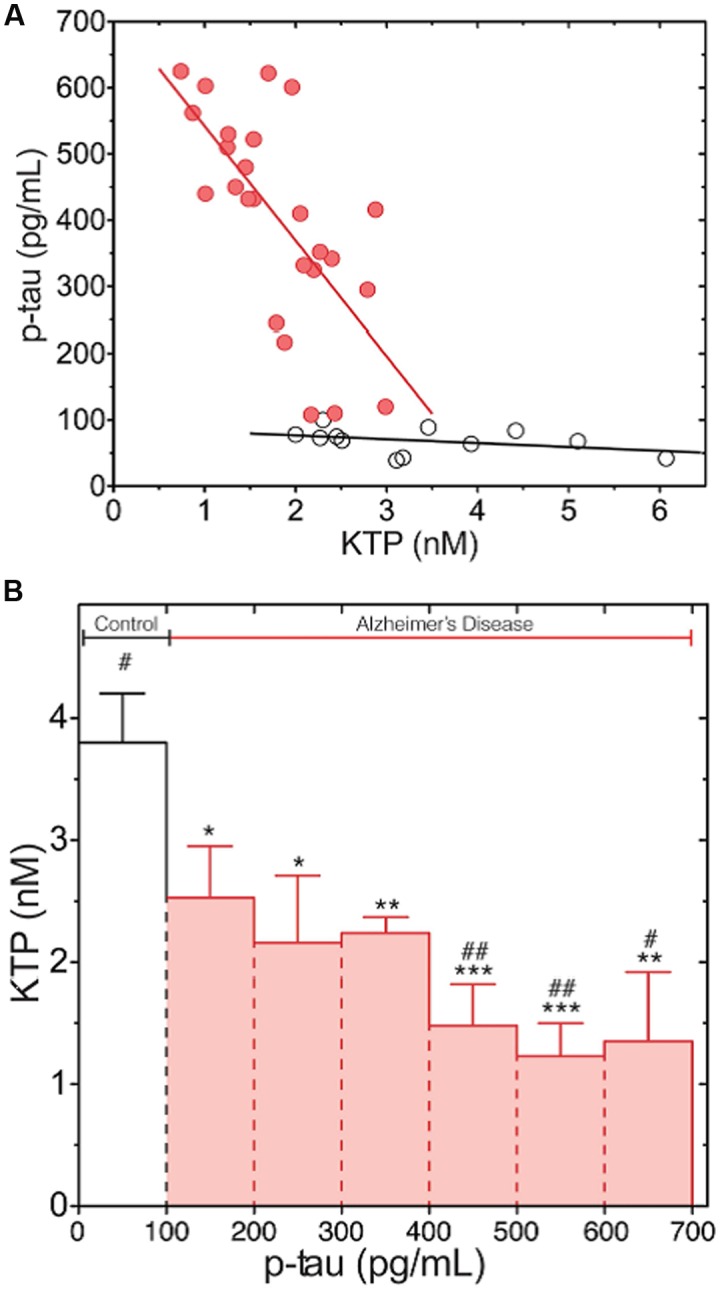
**Phosphorylated-tau (p-Tau) levels dependence on KTP concentration both in AD (red) and normal group (white).** **(A)** Complete data set with individual values (linear regression line plotted as a guide to the eye). **(B)** The data set presented in **(A)** was clustered in classes according to regular intervals of amplitude of 100 pg/mL of p-tau concentration and averaged for KTP concentration (error bars: standard error). The average KTP value of all classes was compared to control (^∗^*p* < 0.04, ^∗∗^*p* < 0.0075, ^∗∗∗^*p* < 0.0006) or the 100- to 200-pg/mL p-tau class (^#^*p* < 0.04, ^##^*p* < 0.0046) using the one-way ANOVA with Dunnett’s post-test (Santos et al., 2013).

Owing to the estimated low concentration of KTP in the human CSF (10^-9^ M) (Nishimura et al., 1991) we had to resort to electrospray ionization tandem mass spectrometry (ESI – MS/MS) (Santos et al., 2013). The decreased levels of KTP in AD samples correlate with a disease-specific atrophy of some brain regions meaning damage and loss of neuronal cells, which possibly results in less KTP being produced and its CSF concentration naturally falling in those patients (**Figure 1**). Moreover, we also found an inverse correlation between levels of KTP and of phosphorylated-tau protein (p-tau) (**Figure 2**) (Santos et al., 2013). CSF p-tau reflects the phosphorylation state of tau and the formation of cortical neurofibrillary tangles in the brain (Flaten et al., 2006). As the disease progresses, more neuronal cells are destroyed, p-tau is released and KTP production is impaired (**Figure 1**). These decreased levels of KTP in the brain will probably contribute to a decreased NOS activity (see Mechanism of Action) causing a NO deficit to such a degree that will further promote the neurodegenerative events characteristic of AD. Disruption of NO homeostasis is known to hasten the development of AD (de la Torre and Stefano, 2000).

## Limited Pharmacological Potential of KTP

The majority of studies on KTP following its discovery aimed to unravel the mechanism of action and to evaluate the analgesic effect of this dipeptide. Although the mechanism of action remains an unsolved issue, direct administration of KTP and some analogs peptides into different parts of CNS showed very promising results regarding analgesia (Yajima et al., 1980; Sakurada et al., 1982; Vaught and Chipkin, 1982; Wang et al., 2001). Hereupon, several groups tested the effects following systemic administration (intraperitoneal i.p., intravenous i.v. and oral) but the results have been disappointing. KTP showed only a brief analgesic activity at a high dose of 200 mg/Kg when administered systemically to rodents (Chen et al., 1998).

Blood–brain barrier constitutes the major obstacle to systemically administered drugs to reach CNS due to the tight junctions that link endothelial cells in the brain capillaries and scarcity of receptors. Less than 2% of the drugs developed to treat CNS disorders cross BBB (Pardridge, 2002). Even some small lipophilic molecules that succeed to diffuse through BBB and penetrate into the brain can be exported back to blood stream by efflux pumps, such as *P*-glycoproteins. In addition, several lytic proteins located in the brain capillary endothelial cells surface form an enzymatic barrier to bioactive peptides from blood.

Obviously, some essential molecules, such as amino acids, hexoses and neuropeptides, which do not fulfill the criteria to diffuse passively through BBB need to reach the brain. Therefore there are specific carriers that mediate their transport. Larger molecules, such as proteins (e.g., insulin and transferrin) are transported by saturable transport systems. On the other hand, positively charged molecules (e.g., histones and cationized albumin) use an adsorptive-mediated endocytosis mechanism to enter the brain (Abbott et al., 2010).

At first it was thought KTP inability to cross BBB was related to low affinity of the peptide to lipid membrane (Chen et al., 1998). More recently the hypothesis that KTP can be pumped out by specific transporters has been raised (Jiang et al., 2009; Serrano et al., 2014a,b).

## Development of New KTP Derivatives

Kyotorphin pharmacological potential is limited probably due to its inability to cross BBB and/or susceptibility to enzymatic degradation. In order to overcome these issues while preserving effectiveness as analgesic, several groups including ours have worked in different strategies to modify the original peptide. Some of these strategies deal with (i) chirality (Rybal’chenko et al., 1999; Lopes et al., 2006a), (ii) use of unnatural amino acids and substitution of peptide bonds (Dzimbova et al., 2014; Serrano et al., 2014b), (iii) conjugation with lipophilic groups (Chen et al., 1998; Wang et al., 2001; Lopes et al., 2006b; Ribeiro et al., 2011b; Serrano et al., 2014b), (iv) cationicity improvement (Ribeiro et al., 2011a).

**FIGURE 3:**
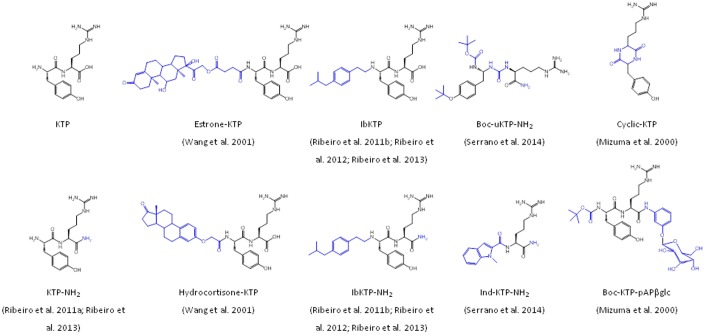
**Chemical structure of KTP derivatives**.

Interestingly, some liphophilic groups added to KTP have by themselves a biological action associated. For instance, Wang et al. (2001) synthesized two distinct KTP derivatives covalently linked to steroids, hydrocortisone (hydrocortisone-KTP) or estrone (estrone-KTP) (**Figure 3**). Unlike KTP, hydrocortisone-KTP and estrone-KTP exhibited good analgesia in the tail-flick test after i.p. administration. These derivatives showed improved pharmacokinetics and pharmacodynamics. Moreover, the researchers suggested that the steroids might be enhancing the KTP effect by increasing the number of its receptors (Wang et al., 2001).

We have extensively studied the analgesic effect of the derivative KTP-NH_2_, which differs from the original peptide by substitution of the carboxylic acid for an amide (**Figure 3**). At physiological pH, this simple chemical modification causes an increase of the net charge of the peptide from +1 to +2. KTP-NH_2_ was tested in acute, sustained and chronic inflammatory and neuropathic pain models following systemic administration (i.p. and oral). At a dosage of 32.3 mg.kg^-1^, KTP-NH_2_ (i.p.) showed an effect comparable to morphine at 5 mg.kg^-1^ in acute pain animal models, meaning the equi-effective dose of KTP-NH_2_ was about fivefold that of morphine. Oral administration required higher dosages to be effective. In chronic pain animal models antinociception induced by KTP-NH_2_ was observed only after a week of daily treatment with 32.3 mg.kg^-1^. In addition, KTP-NH_2_ did not develop resistance unlike morphine, neither jeopardized blood pressure or motor capacity. Accordingly, the peptide showed low affinity to opioid receptors (Ribeiro et al., 2011a).

Other interesting derivatives are IbKTP and IbKTP-NH_2_, which were designed by us to include in their structure a group corresponding to a lipophilic, analgesic and safe non-steroidal anti-inflammatory drug (NSAID), ibuprofen (Ib), covalently linked to the N-terminal of KTP or KTP-NH_2_, respectively (**Figure 3**) (Ribeiro et al., 2011b). In the clinics, combination of different pain killers have been successfully used, e.g., Vicoprofen^®^ (hydrocodone + Ib). Results in acute and chronic pain models showed that both IbKTP and IbKTP-NH_2_, but mainly IbKTP-NH_2_, improved analgesia after systemic administration (Ribeiro et al., 2011b).

**FIGURE 4:**
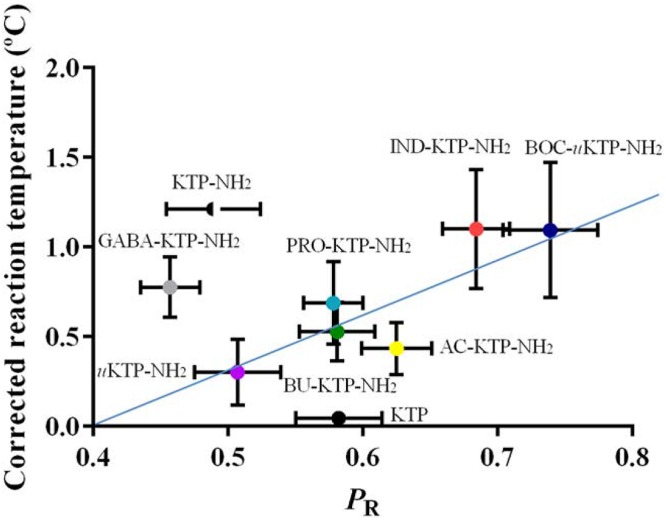
**Correlation between the analgesic efficacy and the relative permeability of KTP derivatives (Serrano et al., 2014b)**.

Recently, eight novel derivatives from KTP and KTP-NH_2_ were synthesized by addition of individual groups at the N-terminus, namely small carbon chains, tert-butyloxycarbonyl (Boc), aminobutyric acid (GABA) or by substitution of the tyrosil residue for an indole moiety. In some cases the peptide bond was substituted by a urea-like bond. The addition of Boc and indolyl groups, but not small carbon chains, increased significantly relative permeability (P_R_) while the peptidomimetics which had the peptide bond substituted by an urea-like bond seemed more resistant to peptidases (Serrano et al., 2014b). Boc-uKTP-NH_2_ and Ind-KTP-NH_2_ (**Figure 3**) were the most promising derivatives, showing a prolonged analgesic effect correlated with higher membrane permeability (**Figure 4**). These two derivatives successfully combined lipophilicity and resistance to enzymatic degradation (Serrano et al., 2014b). Still, some derivatives with lower permeability, such as KTP-NH_2_, have previously demonstrated good analgesic efficacy (Ribeiro et al., 2011a). These results suggest that this particular derivative might have specific transporters to translocate BBB (Serrano et al., 2014b).

Other groups studied other interesting derivatives of KTP, namely a glucose-conjugated KTP and cyclic KTP (**Figure 3**) (Mizuma et al., 2000). Glucose-mediated drug delivery has been a strategy successfully applied to drugs whose final target is in the CNS [for review see (Serrano et al., 2012)]. N-terminal conjugation of p-amino phenyl β-glucoside and C-terminal conjugation of Boc to KTP (Boc-KTP-pAPβglc) (**Figure 3**) enhanced absorption and clearance in the rat intestine and cyclization protected the peptide from proteolytic attack, thereby enhancing enzymatic stability. Cyclic-KTP is suggested as a good analgesic drug candidate to be delivered orally (Mizuma et al., 2000).

### Advantageous Alternatives over Current Opioids

Opium and its alkaloids (e.g., morphine) have been used for centuries as the most powerful centrally acting compounds for the relief of severe acute and chronic pain. However, they also trigger some side-effects such as nausea, constipation, respiratory depression, urinary retention, clouding of consciousness, motor disturbances, tolerance and addiction, which often hamper their widespread use in clinical practice (Fischer et al., 1992). Prolonged admininstration of opioids results in tolerance liability that leads to dose escalation, contributing to an increased incidence and severity of all side-effects. This can lead to discontinued use of the pain medication compromising the quality of life for patients. Thus, the discovery and/or development of potent analgesics that result in effective analgesia with fewer side-effects is greatly needed; and this has long been the holy grail of opioid research (Solé and Barany, 1992; Corbett et al., 2006). In recent years, different approaches have been explored to design and synthesize analogs of naturally occurring opioid peptides (i.e., dermorphin, endomorphins, and enkephalins) as potential substitutes of exogenous opioids for pain relief (Gentilucci, 2004; Janecka et al., 2010; Ribeiro et al., 2011c; Piekielna et al., 2013). Other alternative strategies targeting opioid-receptor have been addressed (Solé and Barany, 1992; Ribeiro et al., 2011c).

KTP-NH_2_ and IbKTP–NH_2_ exhibited analgesic activity comparable to morphine and lower tolerance (Ribeiro et al., 2011a,b). Additionally, no evidences of *in vitro* cytotoxicity or hepatic lesions were detected at effective doses (Ribeiro et al., 2011a). Aiming to validate the pharmaceutical potential of these KTP derivatives as alternative to opioids, further *in vivo* studies were conducted. Hence, for a more detailed pharmacological profiling, both derivatives were studied regarding their side-effects and compared with two clinically relevant opioids, morphine and tramadol (Ribeiro et al., 2013). Particular attention was given to the common opioid-induced side-effects namely on locomotion, micturition, gastrointestinal, and cardiovascular functions (Benyamin et al., 2008). For comparison purposes, morphine and tramadol were selected because morphine remains the gold standard in analgesia (Ramage et al., 1991) while tramadol displays a safer side-effect profile than morphine (Dworkin et al., 2007). In the experimental paradigm, male rats were i.p. injected with a single dose of KTP-NH_2_ (32.3 mg.kg^-1^) or IbKTP-NH_2_ (24.2 mg.kg^-1^) or morphine (5 mg.kg^-1^) or tramadol (10 mg.kg^-1^) before the behavioral/metabolic testing. Doses of KTP derivatives, morphine and tramadol were chosen for inducing comparable analgesia levels in rats (Ribeiro et al., 2013).

Our findings clearly showed that both KTP-derivatives do not cause constipation, in contrast to morphine, and do not induce changes in blood pressure, or in water and food intake, in contrast to tramadol. Despite the fact that KTP–NH_2_ (like tramadol) lowered urine volume this seems to be a minor physiological effect caused by this derivative as no major urinary retention occurred (i.e., increased blood pressure was not observed), and may be exploited as a positive effect in cases of micturition disturbances, i.e., detrusor overactivity. IbKTP-NH_2_ only caused a mild motor impairment that was, however, less harmful than all the severe side-effects induced by tramadol and morphine (Ribeiro et al., 2013).

Overall, KTP derivatives do not trigger the major side-effects intrinsically associated with opioid receptor activation. This correlates with previous findings as direct binding of KTP amidated derivatives to opioid receptors is nearly absent (Ribeiro et al., 2011a,b) similarly to the original KTP molecule (Rackham et al., 1982). Taken together, our data indicates that KTP peptides and opioid drugs exhibit distinct mechanism of action. However, opioid pathways are indirectly involved in KTP peptides mode of action since naloxone decreases the analgesic efficacy of IbKTP-NH_2_ and completely abolishes KTP-NH_2_ analgesic activity (Ribeiro et al., 2011a,b).

Therefore, the strong analgesic activity coupled with the absence of the major side-effects associated to opioids renders both KTP–NH_2_ and IbKTP–NH_2_ as potential advantageous alternatives over current opioids.

## KTP Derivatives Beyond Analgesia

### As Antimicrobial Agents

Antimicrobial peptides represent a promising alternative to conventional antibiotics to fight resistant pathogens because development of resistance is not so effective. They are generally short amphiphilic cationic peptides with high affinity to negatively charged bacterial membranes. One possible mode of action, among others, is membrane disruption caused by peptide insertion into the bacterial membrane, short peptides having higher activity (Lopes-Ferreira et al., 2002; Catiau et al., 2011). Catiau et al. (2011) found that the tripeptide L-lysine-L-tyrosine-L-arginine (KYR) has antimicrobial activity. Since KTP (YR, net charge +1) does not have antimicrobial activity, the positive charge of lysine is of key importance. KTP (net charge: +1), KTP-NH_2_ (+2), IbKTP-NH_2_ (+1) and IbKTP (0) were tested against Gram-negative *Escherichia coli* (*E. coli*) and Gram-positive *Staphylococcus aureus* (*S. aureus*). (Ribeiro et al., 2012). All derivatives were inactive against *E. coli* up to 100 μM but they were active against *S. aureus*, with the exception of KTP. Details of surface structure alteration induced by KTP derivatives (10 μM) in *S. aureus* were obtained by atomic force microscopy (AFM). KTP-NH_2_ and IbKTP-NH_2_ induced shape alterations, unlike KTP and IbKTP. Membrane blebbing and disruption were more evident for KTP-NH_2_. Ib-containing derivatives also interact with red blood cells (RBCs) outer monolayer changing the typical disk shape with uniform borders to spiky boundaries. This shape is known as echinocyte and is reversible. Regardless these changes all derivatives were virtually not toxic to RBCs (Ribeiro et al., 2012).

### Neuroprotective Potential in Cerebral Hypoperfusion Rat Model

In the last two decades, intensive efforts to develop disease-modifying drugs were made to counteract the progression of AD (Flaten et al., 2006). Moreover, increasing attention has been dedicated to neuropeptides in the discovery of new drug targets for the treatment of nervous-system disorders (Hokfelt et al., 2003). Actually, some neuropeptides are densely localized in cognition-related brain regions and play an important role in dementia-associated pathophysiological mechanisms (Borbely et al., 2013).

Over the last decade, some authors hypothesized that KTP has neuromodulating and neuroprotective properties using animal models of cerebral resuscitation (after clinical death) (Nazarenko et al., 1999) and of epilepsy (i.e., picrotoxin- or pentylenetetrazole-induced seizures) (Godlevsky et al., 1995; Bocheva and Dzambazova-Maximova, 2004). In addition, there is consistent evidence about the protective role of NSAIDs, particularly Ib, against neurodegeneration and to reduce the risk of developing AD or Parkinson’s (Asanuma et al., 2001; Chen et al., 2005; Wilkinson et al., 2012). Hence, the potential of a drug candidate (namely KTP) comprising NSAID-based therapy to achieve a clinical benefit would be tremendous. In view of these facts and our recent clinical studies (see KTP as a Promising Biomarker in Alzheimer’s Disease) the relevance of testing our improved KTP derivatives in dementia progression became obvious.

Therefore, KTP-NH_2_ and IbKTP-NH_2_ were recently studied for their ability in post-ischemia to ameliorate cognitive deficits induced by chronic brain hypoperfusion (Sá Santos et al., 2016). Cerebrovascular hypoperfusion is known to be a prominent risk in the development of neurological dysfunction and dementia. In rats, permanent 2VO produces a lasting and reliable reduction of cerebral blood flow, which leads to a progressive neuropathological damage in the hippocampus (particularly its CA1 subfield), learning and memory impairments as it occurs in AD (Farkas et al., 2007).

Our study included rats subjected to permanent global ischemia via 2VO-surgery (2VO-animals) and sham-operated animals (surgery without carotid artery ligation: control group). In the experimental paradigm, 2VO-animals were treated with KTP-NH_2_ (32.3 mg.kg^-1^) or IbKTP-NH_2_ (24.2 mg.kg^-1^) at weeks 2 and 5 post-surgery (single i.p. dose/day for 7 days) (Sá Santos et al., 2016). From a therapeutic perspective, it was of interest to assess their effectiveness after the onset of ischemic injury. Selected doses of KTP-derivatives were based on our previous studies (Ribeiro et al., 2011a,b, 2013).

Following treatment regimen, motor and spatial memory functions were evaluated using the open-field test and two-trial recognition Y-maze task, respectively. Evidences support a direct correlation between cerebral hypoperfusion-induced memory impairments and damage in CA1 pyramidal neurons (De Jong et al., 1999; Farkas et al., 2007; Cechetti et al., 2012). So, we also evaluated hippocampal CA1 integrity through immunohistochemistry protocols (Sá Santos et al., 2016).

Albeit ischemic injury can affect brain regions linked to motor function (i.e., cortex and neocortex), in our study there was no obvious signs of locomotion deficits in 2VO-operated animals (Sá Santos et al., 2016), similar to what has been reported for this experimental model (Farkas et al., 2007; Cechetti et al., 2012). Our findings clearly showed that both KTP-derivatives improved memory deficits of 2VO-animals and prevented CA1 neuronal injury (Sá Santos et al., 2016). Detailed mechanisms underlying their neuroprotective properties are still unknown. However, IbKTP-NH_2_ was the more effective derivative in restoring normal memory function; the presence of NSAID ibuprofen may mitigate some neuroinflammatory events in 2VO-ischemic brain.

### As Anti-Inflammatory Agents

Pain and inflammation are distinct physiological processes but they are frequently associated. Thus, the development of a single drug that could target pain and inflammation simultaneously would be ideal. We used an IVM approach to evaluate the pro- or anti-inflammatory effect of a topical application of KTP (96 μM), KTP-NH_2_ (96 μM), IbKTP (96 μM) and IbKTP-NH_2_ (96 μM) on the cremaster muscle using a rodent model of LPS-induced inflammation (Conceição et al., 2016). We have previously shown that Ib moiety is a enhancer of KTP analgesic action (Ribeiro et al., 2011b). Accordingly, we expected that KTP could also be an enhancer of Ib anti-inflammatory action.

Our data showed that KTP and its analogs did not cause damage on microcirculation. In addition they decreased the number of rolling and adherent leukocytes induced by LPS. This result might be explained by the ability of KTP analogs to bind/perturb LPS micelles, as shown by isothermal titration calorimetry studies, probably contributing to LPS aggregation and subsequent elimination (Conceição et al., 2016). Since KTP did not bind to LPS, the production of NO is the proposed anti-inflammatory mechanism. This is supported by the already discussed mechanism for KTP-induced analgesia that originates L-Arg, a well-known substrate for NOS that ultimately originates NO as product (see Mechanism of Action).

More recently, D-Tyr-L-Arg-NH_2_ (KTP-NH_2_-DL, 96 μM) also decreased the number of rolling leukocytes in a murine model of inflammation induced by LPS, but did not reveal a significant analgesic activity in the hot plate test (Perazzo et al., 2016). This KTP analog and others analyzed in the same study seem to have an action on the endothelium.

## Concluding Remarks

Pain is a huge social and economic problem. In the half last century, innovation in the field of analgesic drug has been scarce. An increased interest has been directed toward peptides as future pain-killers. KTP gathers an interesting and wide set of biological activities; among them, analgesia is by far the most studied. In order to make this peptide more attractive from a pharmacological perspective, several chemical modifications have been made to the original molecule. This strategy has been successful so far and new advantageous properties have emerged from these derivatives, creating a new class of molecules with an increased pharmacological value.

## Author Contributions

JP, MC, and SS listed, have made substantial contribution to the writing and critical revision of the manuscript, and approved it for publication.

## Conflict of Interest Statement

The authors declare that the research was conducted in the absence of any commercial or financial relationships that could be construed as a potential conflict of interest.

## Abbreviations

2VO: bilateral common carotid artery occlusion
AMPs: antimicrobial peptides
Ib: ibuprofen
IVM: intravital microscopy
KTP: kyotorphin
KTPr: KTP receptor
KTP-NH_2_: KTP-amide
LPS: lipopolysaccharide
NO: nitric oxide
NOS: nitric oxide synthetase
PEPT2: peptide transporter 2
PLC: phospholipase C
*P*_R_: relative permeability
SP: substance P

## References

[B1] Abbott N. J. Patabendige A. A. K. Dolman D. E. M. Yusof S. R. Begley D. J. (2010). Structure and function of the blood-brain barrier. *Neurobiol. Dis.* 37 13–25. 10.1016/j.nbd.2009.07.030 19664713

[B2] Akasaki K. Nakamura A. Shiomi H. Tsuji H. (1991). Identification and characterization of two distinct kyotorphin-hydrolyzing enzymes in rat brain. *Neuropeptides* 20 103–107. 10.1016/0143-4179(91)90059-R 1798437

[B3] Akasaki K. Tsuji H. (1991). An enkephalin-degrading aminopeptidase from rat brain catalyzes the hydrolysis of a neuropeptide, kyotorphin (L-Tyr-L-Arg). *Chem. Pharm. Bull. (Tokyo)* 39 1883–1885. 10.1248/cpb.39.1883 1777940

[B4] Akasaki K. Yoshimoto H. Nakamura A. Shiomi H. Tsuji H. (1995). Purification and characterization of a major kyotorphin-hydrolyzing peptidase of rat brain. *J. Biochem.* 117 897–902. 7592556 10.1093/oxfordjournals.jbchem.a124793

[B5] Albericio F. Kneib-Cordonier N. Biancalana S. Gera L. Masada R. I. Hudson D. (1990). Preparation and application of the 5-(4-(9-fluorenylmethyloxycarbonyl)aminomethyl-3,5-dimethoxyphenoxy)-valeric acid (PAL) handle for the solid-phase synthesis of C-terminal peptide amides under mild conditions. *J. Org. Chem.* 55 3730–3743. 10.1021/jo00299a011

[B6] Araque A. Parpura V. Sanzgiri R. P. Haydon P. G. (1999). Tripartite synapses: glia, the unacknowledged partner. *Trends Neurosci.* 22 208–215. 10.1016/s0166-2236(98)01349-6 10322493

[B7] Asanuma M. Nishibayashi-Asanuma S. Miyazaki I. Kohno M. Ogawa N. (2001). Neuroprotective effects of non-steroidal anti-inflammatory drugs by direct scavenging of nitric oxide radicals. *J. Neurochem.* 76 1895–1904. 10.1046/j.1471-4159.2001.00205.x 11259508

[B8] Benyamin R. Trescot A. M. Datta S. Buenaventura R. Adlaka R. Sehgal N. (2008). Opioid complications and side effects. *Pain Physician* 11 2(Suppl.), S105–S120. 18443635

[B9] Berger U. V. Hediger M. A. (1999). Distribution of peptide transporter PEPT2 mRNA in the rat nervous system. *Anat. Embryol. (Berl)* 199 439–449. 10.1007/s004290050242 10221455

[B10] Bocheva A. I. Dzambazova-Maximova E. B. (2004). Effects of kyotorphin and analogues on nociception and pentylenetetrazole seizures. *Folia Med. (Plovdiv)* 46 40–44. 15362813

[B11] Borbely E. Scheich B. Helyes Z. (2013). Neuropeptides in learning and memory. *Neuropeptides* 47 439–450. 10.1016/j.npep.2013.10.012 24210137

[B12] Borsook D. (2012). Neurological diseases and pain. *Brain* 135(Pt 2), 320–344. 10.1093/brain/awr271 22067541 PMC3281476

[B13] Bronnikov G. Dolgacheva L. Zhang S. J. Galitovskaya E. Kramarova L. Zinchenko V. (1997). The effect of neuropeptides kyotorphin and neokyotorphin on proliferation of cultured brown preadipocytes. *FEBS Lett.* 407 73–77. 10.1016/S0014-5793(97)00298-6 9141484

[B14] Catiau L. Traisnel J. Delval-Dubois V. R. Chihib N.-E. Guillochon D. Nedjar-Arroume N. M. (2011). Minimal antimicrobial peptidic sequence from hemoglobin alpha-chain: KYR. *Peptides* 32 633–638. 10.1016/j.peptides.2010.12.016 21262306

[B15] Cechetti F. Pagnussat A. S. Worm P. V. Elsner V. R. Ben J. da Costa M. S. (2012). Chronic brain hypoperfusion causes early glial activation and neuronal death, and subsequent long-term memory impairment. *Brain Res. Bull.* 87 109–116. 10.1016/j.brainresbull.2011.10.006 22040859

[B16] Chen H. Jacobs E. Schwarzschild M. A. McCullough M. L. Calle E. E. Thun M. J. (2005). Nonsteroidal antiinflammatory drug use and the risk for Parkinson’s disease. *Ann. Neurol.* 58 963–967. 10.1002/ana.20682 16240369

[B17] Chen P. Bodor N. Wu W. M. Prokai L. (1998). Strategies to target kyotorphin analogues to the brain. *J. Med. Chem.* 41 3773–3781. 10.1021/jm970715l 9748352

[B18] Cole L. J. Farrell M. J. Duff E. P. Barber J. B. Egan G. F. Gibson S. J. (2006). Pain sensitivity and fMRI pain-related brain activity in Alzheimer’s disease. *Brain* 129(Pt 11), 2957–2965. 10.1093/brain/awl228 16951408

[B19] Conceição K. Magalhães P. R. Campos S. R. R. Domingues M. M. Ramu V. G. Michalek M. (2016). The anti-inflammatory action of the analgesic kyotorphin neuropeptide derivatives: insights of a lipid-mediated mechanism. *Amino Acids* 48 307–318. 10.1007/s00726-015-2088-9 26347373

[B20] Corbett A. D. Henderson G. McKnight A. T. Paterson S. J. (2006). 75 years of opioid research: the exciting but vain quest for the Holy Grail. *Br. J. Pharmacol.* 147(Suppl. 1), S153–S162. 10.1038/sj.bjp.0706435 PMC1760732 16402099

[B21] De Jong G. I. Farkas E. Stienstra C. M. Plass J. R. Keijser J. N. de la Torre J. C. (1999). Cerebral hypoperfusion yields capillary damage in the hippocampal CA1 area that correlates with spatial memory impairment. *Neuroscience* 91 203–210. 10.1016/S0306-4522(98)00659-9 10336071

[B22] de la Torre J. C. Stefano G. B. (2000). Evidence that Alzheimer’s disease is a microvascular disorder: the role of constitutive nitric oxide. *Brain Res. Brain Res. Rev* 34 119–136. 10.1016/S0165-0173(00)00043-6 11113503

[B23] Dieck S. T. Heuer H. Ehrchen J. Otto C. Bauer K. (1999). The peptide transporter PepT2 is expressed in rat brain and mediates the accumulation of the fluorescent dipeptide derivative beta-Ala-Lys-Nepsilon-AMCA in astrocytes. *Glia* 25 10–20. 10.1002/(SICI)1098-1136(19990101)25:1<10::AID-GLIA2>3.0.CO;2-Y 9888294

[B24] Dworkin R. H. O’Connor A. B. Backonja M. Farrar J. T. Finnerup N. B. Jensen T. S. (2007). Pharmacologic management of neuropathic pain: evidence-based recommendations. *Pain* 132 237–251. 10.1016/j.pain.2007.08.033 17920770

[B25] Dzambazova E. B. (2010). The unique brain dipeptide kyotorphin - from discovery to nowadays. *J. Biomed. Clin. Res.* 3 3–11.

[B26] Dzimbova T. Bocheva A. Pajpanova T. (2014). Kyotorphin analogues containing unnatural amino acids: synthesis, analgesic activity and computer modeling of their interactions with m-receptor. *Med. Chem. Res.* 23 3694–3704. 10.1007/s00044-014-0953-9

[B27] Farkas E. Luiten P. G. Bari F. (2007). Permanent, bilateral common carotid artery occlusion in the rat: a model for chronic cerebral hypoperfusion-related neurodegenerative diseases. *Brain Res. Rev.* 54 162–180. 10.1016/j.brainresrev.2007.01.003 17296232

[B28] Fischer P. M. Retson K. V. Tyler M. I. Howden M. E. (1992). Application of arylsulphonyl side-chain protected arginines in solid-phase peptide synthesis based on 9-fluorenylmethoxycarbonyl amino protecting strategy. *Int. J. Pept. Protein Res.* 40 19–24. 10.1111/j.1399-3011.1992.tb00100.x 1428537

[B29] Flaten G. E. Dhanikula A. B. Luthman K. Brandl M. (2006). Drug permeability across a phospholipid vesicle based barrier: a novel approach for studying passive diffusion. *Eur. J. Pharm. Sci.* 27 80–90. 10.1016/j.ejps.2005.08.007 16246536

[B30] Fujita T. Kishida T. Okada N. Ganapathy V. Leibach F. H. Yamamoto A. (1999). Interaction of kyotorphin and brain peptide transporter in synaptosomes prepared from rat cerebellum: implication of high affinity type H+/peptide transporter PEPT2 mediated transport system. *Neurosci. Lett.* 271 117–120. 10.1016/S0304-3940(99)00540-6 10477116

[B31] Gentilucci L. (2004). New trends in the development of opioid peptide analogues as advanced remedies for pain relief. *Curr. Top. Med. Chem.* 4 19–38. 10.2174/1568026043451663 14754374

[B32] Godlevsky L. S. Shandra A. A. Mikhaleva I. I. Vastyanov R. S. Mazarati A. M. (1995). Seizure-protecting effects of kyotorphin and related peptides in an animal model of epilepsy. *Brain Res. Bull.* 37 223–226. 10.1016/0361-9230(94)00274-5 7627564

[B33] Hampel H. Frank R. Broich K. Teipel S. J. Katz R. G. Hardy J. (2010). Biomarkers for Alzheimer’s disease: academic, industry and regulatory perspectives. *Nat. Rev. Drug Discov.* 9 560–574. 10.1038/nrd3115 20592748

[B34] Harima A. Shimizu H. Takagi H. (1991). Analgesic effect of L-arginine in patients with persistent pain. *Eur. Neuropsychopharmacol.* 1 529–533. 10.1016/0924-977X(91)90006-G 1822318

[B35] Hazato T. Kase R. Ueda H. Takagi H. Katayama T. (1986). Inhibitory effects of the analgesic neuropeptides kyotorphin and neo-kyotorphin on enkephalin-degrading enzymes from monkey brain. *Biochem. Int.* 12 379–383. 3011001

[B36] Hokfelt T. Bartfai T. Bloom F. (2003). Neuropeptides: opportunities for drug discovery. *Lancet Neurol.* 2 463–472. 10.1016/S1474-4422(03)00482-4 12878434

[B37] Ignat’ev D. A. Vorob’ev V. V. Ziganshin R. (1998). Effects of a number of short peptides isolated from the brain of the hibernating ground squirrel on the EEG and behavior in rats. *Neurosci. Behav. Physiol.* 28 158–166. 10.1007/BF02461962 9604218

[B38] Inoue M. Nakayamada H. Tokuyama S. Ueda H. (1997). Peripheral non-opioid analgesic effects of kyotorphin in mice. *Neurosci. Lett.* 236 60–62. 10.1016/S0304-3940(97)00760-X 9404952

[B39] Inoue M. Yamada T. Ueda H. (1999). Low dose of kyotorphin (tyrosine-arginine) induces nociceptive responses through a substance P release from nociceptor endings. *Brain Res. Mol. Brain Res.* 69 302–305. 10.1016/S0169-328X(99)00133-3 10366753

[B40] Janecka A. Perlikowska R. Gach K. Wyrebska A. Fichna J. (2010). Development of opioid peptide analogs for pain relief. *Curr. Pharm. Des.* 16 1126–1135. 10.2174/138161210790963869 20030621

[B41] Janicki P. K. Lipkowski A. W. (1983). Kyotorphin and D-kyotorphin stimulate Met-enkephalin release from rat striatum in vitro. *Neurosci. Lett.* 43 73–77. 10.1016/0304-3940(83)90131-3 6142436

[B42] Jiang H. Hu Y. Keep R. F. Smith D. E. (2009). Enhanced antinociceptive response to intracerebroventricular kyotorphin in Pept2 null mice. *J. Neurochem.* 109 1536–1543. 10.1111/j.1471-4159.2009.06090.x 19383084 PMC2898572

[B43] Kawabata A. Muguruma H. Tanaka M. Takagi H. (1996). Kyotorphin synthetase activity in rat adrenal glands and spinal cord. *Peptides* 17 407–411. 10.1016/0196-9781(96)00026-5 8735966

[B44] Kawabata A. Nishimura Y. Takagi H. (1992). l-Leucyl-l-arginine, naltrindole and d-arginine block antinociception elicited by l-arginine in mice with carrageenin-induced hyperalgesia. *Br. J. Pharmacol.* 107 1096–1101. 10.1111/j.1476-5381.1992.tb13413.x 1467831 PMC1907944

[B45] Kawabata A. Umeda N. Takagi H. (1993). L-arginine exerts a dual role in nociceptive processing in the brain: involvement of the kyotorphin-Met-enkephalin pathway and NO-cyclic GMP pathway. *Br. J. Pharmacol.* 109 73–79. 10.1111/j.1476-5381.1993.tb13533.x 8388303 PMC2175590

[B46] Kolaeva S. G. Semenova T. P. Santalova I. M. Moshkov D. A. Anoshkina I. A. Golozubova V. (2000). Effects of L-thyrosyl - L-arginine (kyotorphin) on the behavior of rats and goldfish. *Peptides* 21 1331–1336. 10.1016/S0196-9781(00)00275-8 11072119

[B47] Liu W. Liang R. Ramamoorthy S. Fei Y. J. Ganapathy M. E. Hediger M. A. (1995). Molecular cloning of PEPT 2, a new member of the H+/peptide cotransporter family, from human kidney. *Biochim. Biophys. Acta* 1235 k461–466. 10.1016/0005-2736(95)80036-F 7756356

[B48] Lopes S. C. Fedorov A. Castanho M. A. (2006a). Chiral recognition of D-kyotorphin by lipidic membranes: relevance toward improved analgesic efficiency. *Chem. Med. Chem.* 1 723–728. 10.1002/cmdc.200600096 16902926

[B49] Lopes S. C. Soares C. M. Baptista A. M. Goormaghtigh E. Cabral B. J. Castanho M. A. (2006b). Conformational and orientational guidance of the analgesic dipeptide kyotorphin induced by lipidic membranes: putative correlation toward receptor docking. *J. Phys. Chem. B* 110 3385–3394. 10.1021/jp053651w 16494353

[B50] Lopes-Ferreira M. Moura-da-Silva A. M. Piran-Soares A. A. Angulo Y. Lomonte B. Gutierrez J. M. (2002). Hemostatic effects induced by *Thalassophryne nattereri* fish venom: a model of endothelium-mediated blood flow impairment. *Toxicon* 40 1141–1147. 10.1016/S0041-0101(02)00114-9 12165317

[B51] Machuqueiro M. Baptista A. M. (2007). The pH-dependent conformational states of kyotorphin: a constant-pH molecular dynamics study. *Biophys. J.* 92 1836–1845. 10.1529/biophysj.106.092445 17172294 PMC1861802

[B52] Mizuma T. Koyanagi A. Awazu S. (2000). Intestinal transport and metabolism of glucose-conjugated kyotorphin and cyclic kyotorphin: metabolic degradation is crucial to intestinal absorption of peptide drugs. *Biochim. Biophys. Acta* 1475 90–98. 10.1016/S0304-4165(00)00051-9 10806343

[B53] Nazarenko I. V. Zvrushchenko M. Volkov A. V. Kamenskii A. A. Zaganshin R. (1999). Functional-morphologic evaluation of the effect of the regulatory peptide kyotorphin on the status of the CNS in the post-resuscitation period. *Patol. Fiziol. Eksp. Ter.* 2 31–33. 10379182

[B54] Nishimura K. Kaya K. Hazato T. Ueda H. Satoh M. Takagi H. (1991). Kyotorphin like substance in human cerebrospinal fluid of patients with persistent pain. *Masui* 40 1686–1690. 1766121

[B55] Orawski A. Simmons W. (1992). Dipeptidase activities in rat brain synaptosomes can be distinguished on the basis of inhibition by bestatin and amastatin: identification of a kyotorphin (Tyr-Arg)-degrading enzyme. *Neurochem. Res.* 17 817–820. 10.1007/bf00969018 1641064

[B56] Pardridge W. M. (2002). Why is the global CNS pharmaceutical market so under-penetrated? *Drug Discov. Today* 7 5–7. 10.1016/S1359-6446(01)02082-7 11790589

[B57] Perazzo J. Lopes-Ferreira M. Sá Santos S. Serrano I. Pinto A. Lima C. (2016). Endothelium-mediated action of analogues of the endogenous neuropeptide kyotorphin (tyrosil-arginine): mechanistic insights from permeation and effects on microcirculation. *ACS Chem. Neurosci.* 7 1130–1140. 10.1021/acschemneuro.6b00099 27244291

[B58] Piekielna J. Perlikowska R. Gach K. Janecka A. (2013). Cyclization in opioid peptides. *Curr. Drug Targets* 14 798–816. 10.2174/1389450111314070008 23621510

[B59] Pieper M. J. van Dalen-Kok A. H. Francke A. L. van der Steen J. T. Scherder E. J. Husebo B. S. (2013). Interventions targeting pain or behaviour in dementia: a systematic review. *Ageing Res. Rev.* 12 1042–1055. 10.1016/j.arr.2013.05.002 23727161

[B60] Rackham A. Wood P. L. Hudgin R. L. (1982). Kyotorphin (tyrosine-arginine): further evidence for indirect opiate receptor activation. *Life Sci.* 30 1337–1342. 10.1016/0024-3205(82)90017-0 6283289

[B61] Ramage R. Green J. Blake A. J. (1991). An acid labile arginine derivative for peptide synthesis: NG-2,2,5,7,8-pentamethylchroman-6-sulphonyl-L-arginine. *Tetrahedron* 47 6353–6370. 10.1016/S0040-4020(01)86564-9

[B62] Raskind M. A. Peskind E. R. Lampe T. H. Risse S. C. Taborsky G. J. Jr. Dorsa D. (1986). Cerebrospinal fluid vasopressin, oxytocin, somatostatin, and beta-endorphin in Alzheimer’s disease. *Arch. Gen. Psychiatry* 43 382–388. 10.1001/archpsyc.1986.01800040092013 2869744

[B63] Ribeiro M. M. Pinto A. Pinto M. Heras M. Martins I. Correia A. (2011a). Inhibition of nociceptive responses after systemic administration of amidated kyotorphin. *Br. J. Pharmacol.* 163 964–973. 10.1111/j.1476-5381.2011.01290.x 21366550 PMC3130928

[B64] Ribeiro M. M. Pinto A. R. Domingues M. M. Serrano I. Heras M. Bardaji E. R. (2011b). Chemical conjugation of the neuropeptide kyotorphin and ibuprofen enhances brain targeting and analgesia. *Mol. Pharm.* 8 1929–1940. 10.1021/mp2003016 21830793

[B65] Ribeiro M. M. B. Serrano I. D. Sá Santos S. (2011c). “Turning Endogenous Peptides Into New Analgesics: The Example of Kyotorphin Derivatives,” in *Peptide Drug Discovery and Development: Translational Research in Academia and Industry*, 1st Edn, eds Castanho M. Santos N. (Weinheim: WILEY-VCH Verlag GmbH & Co. KGaA), 171–188.

[B66] Ribeiro M. M. Sá Santos S. Sousa D. S. Oliveira M. Santos S. M. Heras M. (2013). Side-effects of analgesic kyotorphin derivatives: advantages over clinical opioid drugs. *Amino Acids* 45 171–178. 10.1007/s00726-013-1484-2 23471674

[B67] Ribeiro M. M. B. Franquelim H. G. Torcato I. S. M. Ramu V. G. Heras M. Bardaji E. R. (2012). Antimicrobial properties of analgesic kyotorphin peptides unraveled through atomic force microscopy. *Biochem. Biophys. Res. Commun.* 420 676–679. 10.1016/j.bbrc.2012.03.065 22450328

[B68] Rybal’chenko V. K. Ostrovskaya G. V. Poralo I. V. Rybal’chenko T. V. Mel’nik Y. M. (1999). Membranotropic activity of optical isomers of the neuropeptide kyotorphin and a cardiotonic agent, suphan. *Neurophysiology* 31 223–225. 10.1007/bf02515077

[B69] Sá Santos S. Santos S. M. Pinto A. R. Ramu V. G. Heras M. Bardaji E. (2016). Amidated and ibuprofen-conjugated kyotorphins promote neuronal rescue and memory recovery in cerebral hypoperfusion dementia model. *Front. Aging Neurosci.* 8:1 10.3389/fnagi.2016.00001 PMC4726799 26858637

[B70] Sakurada S. Sakurada T. Jin H. Sato T. Kisara K. Sasaki Y. (1982). Antinociceptive activities of synthetic dipeptides in mice. *J. Pharm. Pharmacol.* 34 750–751. 10.1111/j.2042-7158.1982.tb06218.x 6129313

[B71] Sakurada T. Sakurada S. Watanabe S. Matsumura H. Kisara K. Akutsu Y. (1983). Actions of intracerebroventricular administration of kyotorphin and an analog on thermoregulation in the mouse. *Peptides* 4 859–863. 10.1016/0196-9781(83)90081-5 6424102

[B72] Santos S. Castanho M. (2013). The use of visual analog scales to compare pain between patients with Alzheimer’s disease and patients without any known neurodegenerative disease and their caregivers. *Am. J. Alzheimers Dis. Other Demen.* 29 320–325. 10.1177/1533317513517046 24370623 PMC10852572

[B73] Santos S. M. Garcia-Nimo L. Sá Santos S. Tavares I. Cocho J. A. Castanho M. A. (2013). Neuropeptide kyotorphin (tyrosyl-arginine) has decreased levels in the cerebro-spinal fluid of Alzheimer’s disease patients: potential diagnostic and pharmacological implications. *Front. Aging Neurosci.* 5:68 10.3389/fnagi.2013.00068 PMC3812564 24198785

[B74] Serrano I. D. Freire J. M. Carvalho M. V. Neves M. Melo M. N. Castanho M. A. R. B. (2014a). The Mechanisms and quantification of the selective permeability in transport across biological barriers: the example of kyotorphin. *Mini Rev. Med. Chem.* 14 99–110. 10.2174/1389557514666140123130058 24456269

[B75] Serrano I. D. Ramu V. G. Pinto A. R. T. Freire J. M. Tavares I. Heras M. (2014b). Correlation between membrane translocation and analgesic efficacy in kyotorphin derivatives. *Peptide Sci.* 104 1–10. 10.1002/bip.22580 25363470

[B76] Serrano I. D. Ribeiro M. M. Castanho M. A. (2012). A focus on glucose-mediated drug delivery to the central nervous system. *Mini Rev. Med. Chem.* 12 301–312. 10.2174/138955712799829302 22303945

[B77] Shiomi H. Kuraishi Y. Ueda H. Harada Y. Amano H. Takagi H. (1981). Mechanism of kyotorphin-induced release of Met-enkephalin from guinea pig striatum and spinal cord. *Brain Res.* 221 161–169. 10.1016/0006-8993(81)91070-2 7272759

[B78] Shu C. Shen H. Teuscher N. S. Lorenzi P. J. Keep R. F. Smith D. E. (2002). Role of PEPT2 in peptide/mimetic trafficking at the blood-cerebrospinal fluid barrier: studies in rat choroid plexus epithelial cells in primary culture. *J. Pharmacol. Exp. Ther.* 301 820–829. 10.1124/jpet.301.3.820 12023509

[B79] Solé N. A. Barany G. (1992). Optimization of solid-phase synthesis of [Ala8]-dynorphin-A. *J. Org. Chem.* 57 5399–5403. 10.1021/Jo00046a022

[B80] Summy-Long J. Y. Bui V. Gestl S. Koehler-Stec E. Liu H. Terrell M. L. (1998). Effects of central injection of kyotorphin and L-arginine on oxytocin and vasopressin release and blood pressure in conscious rats. *Brain Res. Bull.* 45 395–403. 10.1016/S0361-9230(97)00341-9 9527014

[B81] Takagi H. (1990). [Physiological and pharmacological actions of a neuroactive dipeptide, kyotorphin, and its precursor, L-arginine, and clinical application]. *Nihon Yakurigaku Zasshi* 96 85–96. 10.1254/fpj.96.3_85 2272541

[B82] Takagi H. Harima A. Shimizu H. (1990). A novel clinical treatment of persistent pain with L-arginine. *Eur. J. Pharmacol.* 183: 1443 10.1016/0014-2999(90)94580-Q

[B83] Takagi H. Shiomi H. Kuraishi Y. Ueda H. (1982). Analgesic dipeptide, L-Tyr-D-Arg (D-kyotorphin) induces Met-enkephalin release from guinea-pig striatal slices. *Experientia* 38 1344–1345. 10.1007/bf01954941

[B84] Takagi H. Shiomi H. Ueda H. Amano H. (1979a). Morphine-like analgesia by a new dipeptide, L-tyrosyl-L-arginine (Kyotorphin) and its analogue. *Eur. J. Pharmacol.* 55 109–111. 10.1016/0014-2999(79)90154-7 436940

[B85] Takagi H. Shiomi H. Ueda H. Amano H. (1979b). A novel analgesic dipeptide from bovine brain is a possible Met-enkephalin releaser. *Nature* 282 410–412. 10.1038/282410a0 228202

[B86] Thakkar S. V. Miyauchi S. Prasad P. D. Ganapathy V. (2008). Stimulation of Na+/Cl–coupled opioid peptide transport system in SK-N-SH cells by L-kyotorphin, an endogenous substrate for H+-coupled peptide transporter PEPT2. *Drug Metab. Pharmacokinet.* 23 254–262. 10.2133/dmpk.23.254 18762712

[B87] Ueda H. Inoue M. (2000). In vivo signal transduction of nociceptive response by kyotorphin (tyrosine-arginine) through Gai- and inositol trisphosphate-mediated Ca^2+^ influx. *Mol. Pharm.* 57 108–115. 10617685

[B88] Ueda H. Inoue M. Weltrowska G. Schiller P. W. (2000). An enzymatically stable kyotorphin analog induces pain in subattomol doses. *Peptides* 21 k717–722. 10.1016/S0196-9781(00)00190-X 10876055

[B89] Ueda H. Matsumoto S. Yoshihara Y. Fukushima N. Takagi H. (1986a). Uptake and release of kyotorphin in rat brain synaptosomes. *Life Sci.* 38 2405–2411. 10.1016/0024-3205(86)90609-0 3724363

[B90] Ueda H. Ming G. Hazato T. Katayama T. Takagi H. (1985). Degradation of kyotoprhin by a purified membrane-bound-aminopeptidase from monkey brain: potentiation of kyotorphin-induced analgesia by a highly effective inhibitor, bestatin. *Life Sci.* 36 1865–1871. 10.1016/0024-3205(85)90160-2 3990513

[B91] Ueda H. Shiomi H. Takagi H. (1980). Regional distribution of a novel analgesic dipeptide kyotorphin (Tyr-Arg) in the rat brain and spinal cord. *Brain Res.* 198 460–464. 10.1016/0006-8993(80)90761-1 7407611

[B92] Ueda H. Yoshihara Y. Fukushima N. Shiomi H. Nakamura A. Takagi H. (1987). Kyotorphin (tyrosine-arginine) synthetase in rat brain synaptosomes. *J. Biol. Chem.* 262 8165–8173. 3597366

[B93] Ueda H. Yoshihara Y. Misawa H. Fukushima N. Katada T. Ui M. (1989). The kyotorphin (tyrosine-arginine) receptor and a selective reconstitution with purified Gi, measured with GTPase and phospholipase C assays. *J. Biol. Chem.* 264 3732–3741. 2537290

[B94] Ueda H. Yoshihara Y. Takagi H. (1986b). A putative met-enkephalin releaser, kyotorphin enhances intracellular Ca^2+^ in the synaptosomes. *Biochem. Biophys. Res. Commun.* 137 897–902. 10.1016/0006-291X(86)91164-2 2425804

[B95] Vaught J. L. Chipkin R. E. (1982). A characterization of kyotorphin (Tyr-Arg)-induced antinociception. *Eur. J. Pharmacol.* 79 167–173. 10.1016/0014-2999(82)90622-7 7047176

[B96] Wang C. Zhao M. Yang J. Peng S. (2001). Synthesis and analgesic effects of kyotorphin-steroid linkers. *Steroids* 66 811–815. 10.1016/S0039-128X(01)00112-X 11576620

[B97] WHO (2012). *Dementia: A Public Health Priority. WHO Library Cataloguing-in-Publication Data*. Geveva: WHO.

[B98] Wilkinson B. L. Cramer P. E. Varvel N. H. Reed-Geaghan E. Jiang Q. Szabo A. (2012). Ibuprofen attenuates oxidative damage through NOX2 inhibition in Alzheimer’s disease. *Neurobiol. Aging* 33 e121–e132. 10.1016/j.neurobiolaging.2010.06.014 PMC2980562 20696495

[B99] Xiang J. Jiang H. Hu Y. Smith D. E. Keep R. F. (2010). Kyotorphin transport and metabolism in rat and mouse neonatal astrocytes. *Brain Res.* 1347 11–18. 10.1016/j.brainres.2010.05.094 20537989 PMC2913889

[B100] Yajima H. Ogawa H. Ueda H. Takagi H. (1980). Studies on peptides. XCIV. Synthesis and activity of kyotorphin and its analogs. *Chem. Pharm. Bull. (Tokyo)* 28 1935–1938. 10.1248/cpb.28.1935 7408060

[B101] Yoshihara Y. Ueda H. Fujii N. Shide A. Yajima H. Satoh M. (1990). Purification of a novel type of calcium-activated neutral protease from rat brain. Possible involvement in production of the neuropeptide kyotorphin from calpastatin fragments. *J. Biol. Chem.* 265 5809–5815. 2318836

